# Microstructural Origin of Magnetic Properties in Grain-Oriented Silicon Steel: Shear Band Angle Control of Intraband Lattice Rotation

**DOI:** 10.3390/ma19214465

**Published:** 2026-10-23

**Authors:** Sifei Cheng, Yuhui Sha, Fang Zhang, Gengsheng Cao, Zhangchen Xue, Liang Zuo

**Affiliations:** Key Laboratory for Anisotropy and Texture of Materials (Ministry of Education), Northeastern University, Shenyang 110819, China; sifei_cheng@163.com (S.C.);

**Keywords:** grain-oriented silicon steel, magnetic properties, shear band, lattice rotation, crystal plasticity, quasi-in situ EBSD, Goss texture

## Abstract

The magnetic properties of grain-oriented silicon steel depend on the sharpness of its Goss texture, which is governed by the density of Goss areas formed in cold-rolling shear bands. This density rises with the intraband lattice rotation rate ω, which is governed by the shear band angle θ through the local shear strain tensor. Reported relationships between θ and the intraband Goss density are inconsistent, and how θ influences ω remains unclear. Slip geometry analysis, crystal plasticity calculations and quasi-in situ electron backscatter diffraction (EBSD) were combined to investigate an Fe-3.0 wt.% Si alloy. The results show that the intraband orientation evolution is retarded by the primary slip system $\left(112\right)\left[\bar{1}\bar{1}1\right]$, most strongly at the retardation plane $\Phi_{r}$ = 35.3° + θ. The {111} matrix accordingly divides into locked, escaped and free regions, depending on whether the orientation reaches the <110>//ND fiber within a finite strain. As θ falls from 35° to 17°, ω of the escaped region rises. Quasi-in situ tracking supported the increase in ω as θ decreases. Despite a smaller shear strain (2.82 vs 3.63), the θ = 25° band crossed the <110>//ND fiber, whereas the θ = 30° band had not yet reached the fiber. θ alters both the slip system activity at the retardation plane and where the orientation crosses it. These results provide a crystallographic basis for controlling intraband Goss density.

## 1. Introduction

Grain-oriented silicon steel is an important soft magnetic material for transformer cores. The central processing objective is to develop a single, sharp Goss {110}<001> texture in the final sheet through cold rolling and secondary recrystallization annealing [1,2,3]. In BCC iron-based alloys, <001> is the easy magnetization direction [4], and the Goss orientation aligns <001> precisely with the magnetization direction in service, i.e., the RD (rolling direction). Consequently, both the magnetic induction and the core loss of the final sheet depend strongly on the sharpness of the Goss texture [5,6,7]. During cold rolling, the <111>//ND (normal direction) deformed matrix has a high Taylor factor and cannot readily accommodate plastic deformation by homogeneous slip. Localized plastic instability, predominantly in the form of shear bands, therefore occurs [8,9]. Shear deformation within the band induces lattice rotation about an axis approximately parallel to TD (transverse direction), moving the intraband orientation from the <111>//ND matrix towards the <110>//ND fiber where the Goss orientation lies [10,11]. The Goss areas thus formed within the shear bands serve as the key Goss “seeds” for abnormal grain growth during secondary recrystallization [12,13,14].

However, a higher density of intraband Goss areas is not necessarily beneficial. An excessive density of Goss nuclei gives rise to orientation pinning during annealing [15,16]. Takajo et al. [17] found that Goss clusters readily form within the shear bands of cold-rolled Fe-3% Si alloy during annealing. They also reported that the low-angle grain boundaries within these clusters (misorientation below 15°) prevent further growth above 750 °C. Liang et al. [18] produced ultra-thin strips from finished grain-oriented silicon steel. They found that the high-induction starting material developed a higher density of intraband Goss areas after cold rolling and formed Goss clusters dominated by low-angle grain boundaries in the early stage of annealing. After secondary recrystallization, the ultra-thin strip obtained from this material exhibited a magnetic induction B_8/50_ (800 A/m, 50 Hz) of only 1.73 T, markedly lower than the 1.92 T obtained from the conventional induction starting material. Paolinelli et al. [19,20] further pointed out that when Goss nuclei are excessively dense, the refinement of the recrystallized grains caused by such dense nucleation raises the core loss. Evidently, too low a density of intraband Goss areas leaves secondary recrystallization short of Goss “seeds”, whereas too high a density suppresses the abnormal growth of Goss grains through orientation pinning. Either case is detrimental to the magnetic properties of the final sheet.

The density of intraband Goss areas is closely related to the shear band angle θ (the angle between the shear band and RD), yet the reported trends remain inconsistent. Ushioda et al. [21] and Song et al. [22] used different thresholds for identifying accurate Goss grains: $\Delta_{mis}$ ≤ 10° and $\Delta_{mis}$ ≤ 15°, where $\Delta_{mis}$ denotes the deviation from ideal Goss. Both studies examined shear bands in the {111}<112> matrix of partially recrystallized cold-rolled Fe-3% Si. They found higher densities of accurate Goss grains in high-angle shear bands (θ = 35° and 29°, respectively) than in low-angle ones (θ = 17°). By contrast, Samajdar et al. [23] ($\Delta_{mis}$ ≤ 5°) reached the opposite conclusion: the density of accurate Goss grains in θ = 17° shear bands was higher than in θ = 37° ones. Notably, the three studies adopted different criteria for accurate Goss grains, which may be a source of the discrepancy. The intraband lattice evolution toward the <110>//ND fiber is essentially a process of lattice rotation. Therefore, the lattice rotation rate ω can be used to characterize the tendency to form intraband Goss areas: a higher ω favors a higher density of intraband Goss areas. Since the intraband lattice rotation is essentially the constitutive response of the deformed matrix to the local shear deformation [24], the shear strain tensor that drives this deformation becomes the key variable governing ω. When the shear band is treated as a plate-like feature perpendicular to the ND–RD plane, this shear strain tensor can be described by the single variable θ [25]. Understanding the relationship between θ and ω is therefore the basis for controlling the density of intraband Goss. However, the studies cited above stop at summarizing the phenomena and have not addressed, at the level of crystal plasticity deformation mechanisms, how θ influences ω.

Accordingly, the present work combines slip geometry analysis, crystal plasticity calculations and quasi-in situ characterization, with θ as the sole variable. The relationship between the shear band angle and the intraband lattice rotation is examined, together with its underlying slip mechanism. The aim is to provide a theoretical basis for the quantitative control of intraband Goss density and, in turn, for the optimization of the magnetic properties of grain-oriented silicon steel.

## 2. Materials and Methods

### 2.1. Material and Quasi-In Situ Characterization

A large initial grain size favors the formation of shear bands [26,27] and facilitates quasi-in situ observation. A hot-rolled Fe-3.0 wt.% Si sheet was therefore used as the starting material, and millimeter-sized initial grains were obtained by high-temperature annealing. To avoid inhibition of grain growth during annealing, the material contained no inhibitor-forming elements such as Mn, S, Al or N. The as-received hot-rolled sheet was 3.6 mm thick and was split at mid-thickness into two 1.8 mm thick halves. This increased the relative fraction of the subsurface region, where shear texture components more readily evolve toward the <111>//ND matrix during subsequent cold rolling. The annealing temperature of 1100 °C was the maximum available furnace temperature. Preliminary trials showed that holding for 30 min at this temperature produced grains of approximately 1 mm. Such grains provide a sufficiently large intragranular region with deformation close to ideal plane strain compression. The 1.8 mm thick sheet was annealed at 1100 °C for 30 min under an argon atmosphere and then water-quenched to freeze the grain structure. Electron backscatter diffraction (EBSD) orientation measurements (step size 8 μm) were then performed on the ND–RD plane of the specimen. A target grain was selected from these measurements for the subsequent rolling. A quasi-in situ characterization scheme was adopted in order to track the orientation evolution of both the matrix and the shear band interiors within the same grain. To ensure that the same area was located at every measurement, microhardness indents were made around the target grain as positioning markers prior to measurement.

A two-step cold-rolling procedure was employed so that the relationship between the shear bands and the deformed matrix could be resolved unambiguously. In the first step, the annealed specimen was rolled to a thickness of 1.08 mm (cumulative true strain 0.51, reduction 40%) in 20 passes of equal true strain increment (≈0.026). The small strain per pass was intended to suppress premature shear band initiation and to keep the deformation within the grain as homogeneous as possible at this stage. After rolling, the orientation of the matrix was measured again (step size 2 μm).

Before the second rolling step, the area containing the target grain was polished to restore a flat surface, so that parallel scratches could be introduced as marker lines by unidirectional grinding with 120-grit abrasive paper. The same specimen was then rolled to 0.81 mm (cumulative true strain 0.8, reduction 55%) in a single pass. The larger strain per pass was intended to trigger shear bands extensively within the matrix. After rolling, the displacement of the marker lines across the shear bands was measured metallographically and used to calculate the local shear strain. The specimen was subsequently held at 650 °C for 30 s in vacuum. This brief recovery treatment relieved the lattice distortion within the bands while avoiding recrystallization, thereby improving the EBSD indexing rate in the subsequent intraband orientation measurements (step size 0.25–0.3 μm).

Throughout the above procedure, EBSD specimens were prepared by electropolishing in a 4 vol.% perchloric acid–ethanol solution at 18 V for 18 s. EBSD orientation measurements were carried out on the electropolished specimens using a Crossbeam-550 scanning electron microscope (Carl Zeiss Microscopy GmbH, Oberkochen, Germany) equipped with a Symmetry S3 detector (Oxford Instruments NanoAnalysis, High Wycombe, UK). An accelerating voltage of 20 kV and a working distance of 12.5 mm were used. EBSD orientation data were analyzed using AZtecCrystal software (Oxford Instruments NanoAnalysis, High Wycombe, UK).

### 2.2. Crystal Plasticity Calculation

At the kinematic level, the finite deformation framework [28,29] was adopted. In this framework, the velocity gradient imposed by the shear band is decomposed into its symmetric part (strain rate $D^{sb}$) and antisymmetric part (rotation rate $\Omega^{sb}$). Since the velocity gradient is the sum of plastic and lattice contributions, these two parts can be expressed as follows:

(1) $$D^{sb}=D^{p}+D^{*},\;\;\Omega^{sb}=\Omega^{p}+\Omega^{*}.$$

The detailed derivation is provided in the Supplementary Materials. In Equation (1), $D^{p}$ and $\Omega^{p}$ denote the plastic strain rate and the plastic spin rate of the material, respectively, while $D^{*}$ and $\Omega^{*}$ denote the elastic strain rate and the spin rate of the lattice. Denoting the unit vectors of the slip direction and the slip plane normal of slip system α in the global coordinate system RD–TD–ND as $b^{\alpha }$ and $n^{\alpha }$, respectively, the Schmid tensor is constructed as

(2) $$M^{\alpha }=b^{\alpha }⊗n^{\alpha }.$$

For the cold rolling of BCC metals, 24 slip systems were included in the calculation (12 of the {110}<111> family and 12 of the {112}<111> family) [30]. $D^{p}$ and $\Omega^{p}$ are then obtained by summing the contributions of the individual slip systems:

(3) $$D^{p}=\sum_{\alpha =1}^{24}{\dot{\gamma }}^{\alpha }M^{\alpha \_s},\;\;\Omega^{p}=\sum_{\alpha =1}^{24}{\dot{\gamma }}^{\alpha }M^{\alpha \_a}.$$

In Equation (3), ${\dot{\gamma }}^{\alpha }$ is the slip rate of slip system α, and $M^{\alpha \_s}$ and $M^{\alpha \_a}$ are the symmetric and antisymmetric parts of $M^{\alpha }$, respectively.

At the constitutive level, the slip rate is described by the viscoplastic flow rule proposed by Hutchinson [31]:

(4) $${\dot{\gamma }}^{\alpha }={\dot{\gamma }}^{0}\cdot sign\left(\frac{\tau^{\alpha }}{g^{\alpha }}\right)\cdot {\left|\frac{\tau^{\alpha }}{g^{\alpha }}\right|}^{m}.$$

In Equation (4), $\tau^{\alpha }$ and $g^{\alpha }$ are the current resolved shear stress and the current strength of slip system α, while the constants m and ${\dot{\gamma }}^{0}$ are the rate sensitivity exponent and the reference slip rate. The macroscopic elastic constitutive relation is established using the Jaumann rate of the Kirchhoff stress τ, denoted $\tau^{\nabla *}$:

(5) $$\tau^{\nabla *}=C:D^{*},$$

where C is the fourth-order elastic stiffness tensor. The rate of change of $\tau^{\alpha }$ is then given by

(6) $${\dot{\tau }}^{\alpha }=M^{\alpha \_s}:\tau^{\nabla *}.$$

At the hardening level, the evolution of the slip system strength is described by the hardening model proposed by Peirce et al. [32]:

(7) $${\dot{g}}^{\alpha }=\sum_{\beta }h_{\alpha \beta }\left|{\dot{\gamma }}^{\beta }\right|.$$

In Equation (7), the hardening modulus $h_{\alpha \beta }$ represents the increment in $g^{\alpha }$ produced by unit slip on slip system β, and is expressed as

(8) $$h_{\alpha \beta }=\left\{\begin{array}{c} qh_{0}{\mathrm{sech}}^{2}\left|\frac{h_{0}\gamma }{\tau_{s}-g_{0}}\right|,\;\alpha =\beta \\ {q_{1}h}_{0}{\mathrm{sech}}^{2}\left|\frac{h_{0}\gamma }{\tau_{s}-g_{0}}\right|,\;\alpha \neq \beta \end{array}\right.,$$

where $h_{0}$, q and $q_{1}$ are the initial hardening modulus and the self- and latent-hardening interaction coefficients, respectively; $g_{0}$ and $\tau_{s}$ are the initial and saturation strengths of the slip systems; and γ is the sum of the accumulated slip over all slip systems:

(9) $$\gamma =\sum_{\alpha =1}^{24}\int_{0}^{t}\left|{\dot{\gamma }}^{\alpha }\right|dt.$$

**Figure 1:**
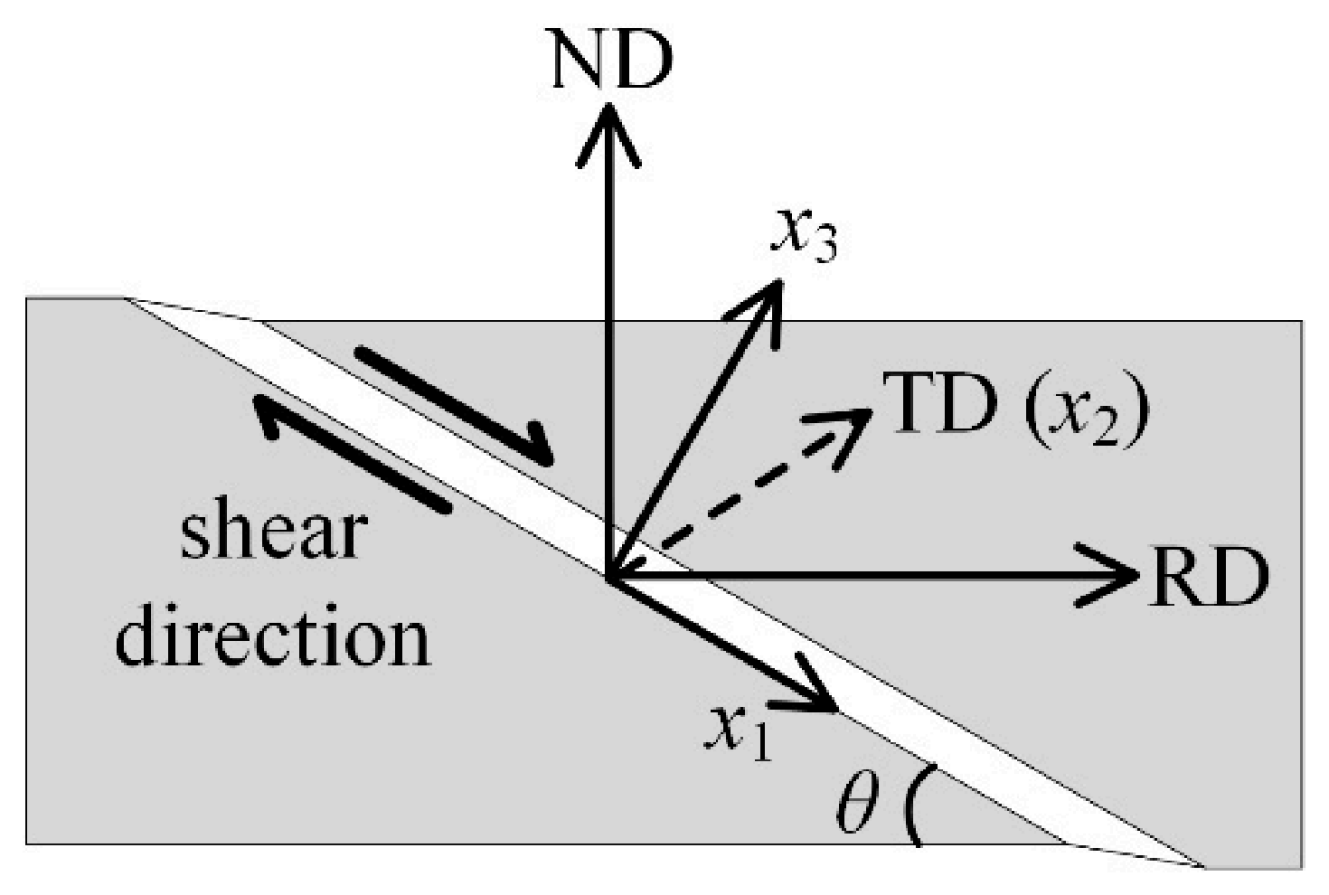
Geometric relationship between the shear band coordinate system *x*_1_–*x*_2_–*x*_3_ and the global coordinate system RD–TD–ND.

As for the boundary conditions, the shear band is treated as a plate-like structure perpendicular to the ND–RD plane and inclined at an angle θ to RD, within which the deformation is simple shear [33,34]. A shear band coordinate system *x*_1_–*x*_2_–*x*_3_ is defined, its relationship to the global coordinate system being shown in Figure 1. Setting the intraband shear strain rate equal to the reference slip rate ${\dot{\gamma }}^{0}$, the strain rate $D^{sb}$ and the rotation rate $\Omega^{sb}$ imposed by the shear band are expressed in the global coordinate system as

(10) $$D^{sb}=\frac{{\dot{\gamma }}^{0}}{2}\left[\begin{array}{ccc} sin2\theta & 0 & cos2\theta \\ 0 & 0 & 0\\ cos2\theta & 0 & -sin2\theta \end{array}\right],\;\;\Omega^{sb}=\frac{{\dot{\gamma }}^{0}}{2}\left[\begin{array}{ccc} 0 & 0 & 1\\ 0 & 0 & 0\\ -1 & 0 & 0 \end{array}\right].$$

On the basis of Equations (4)–(10), ${\dot{\gamma }}^{\alpha }$ is solved using the numerical implementation proposed by Huang [35]. Then, the lattice spin rate $\Omega^{*}$ is obtained through Equations (1)–(3) together with Equation (10). The orientation is then updated according to [36]:

(11) $${\dot{\varphi }}_{1}={-\Omega }_{12}^{*}-{\dot{\varphi }}_{2}\cdot cos\Phi ,$$

(12) $$\dot{\Phi }={-\Omega }_{23}^{*}\cdot cos\varphi_{1}-\Omega_{31}^{*}\cdot sin\varphi_{1},$$

(13) $${\dot{\varphi }}_{2}=\left({-\Omega }_{23}^{*}\cdot sin\varphi_{1}+\Omega_{31}^{*}\cdot cos\varphi_{1}\right)/sin\Phi ,$$

where $\varphi_{1}$, Φ and $\varphi_{2}$ are the Euler angles. The parameter values used in the calculations are listed in Table 1.

### 2.3. Matrix Orientation Region and Expression of the Lattice Rotation Rate

**Figure 2:**
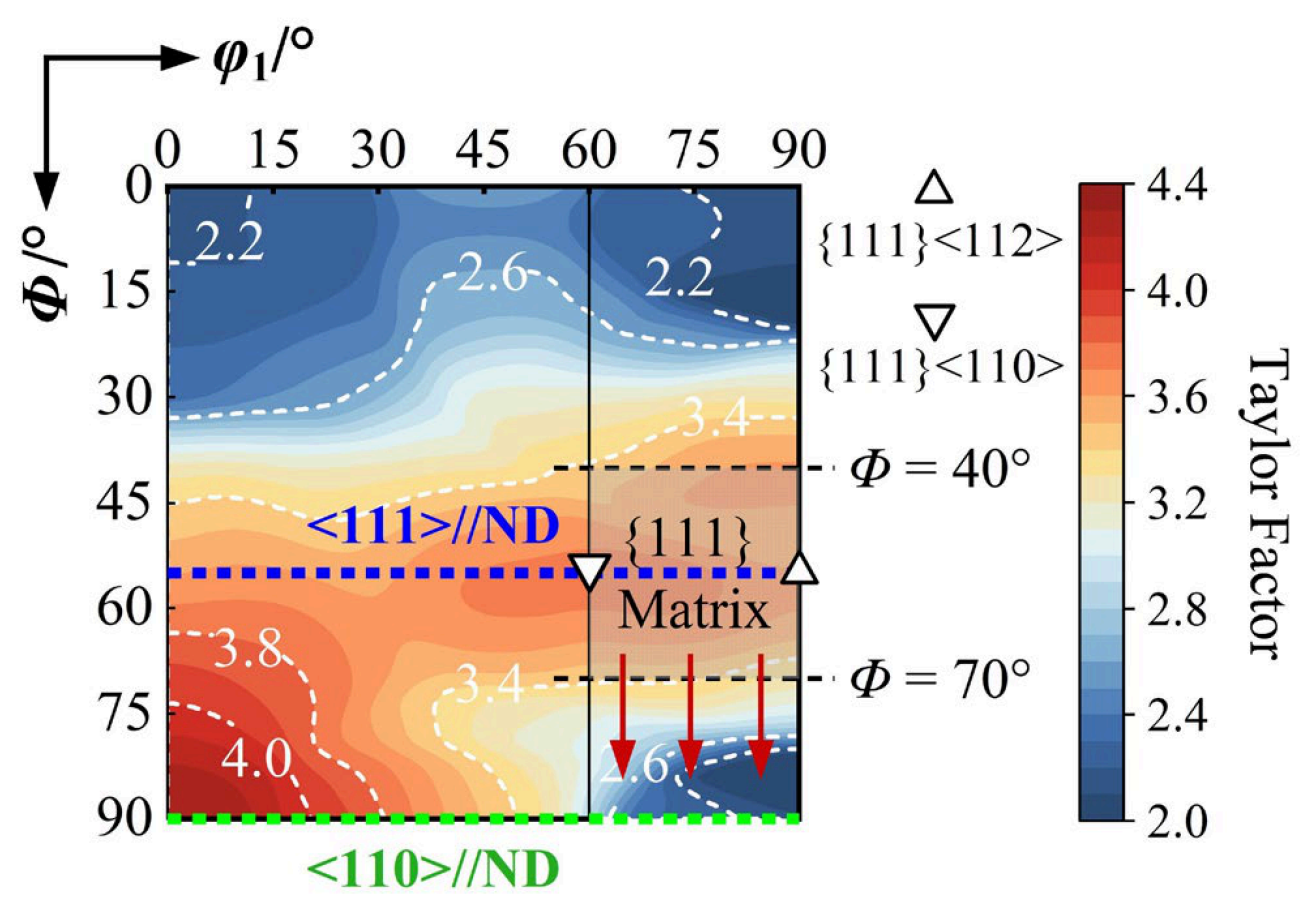
Distribution of the Taylor factor on the $\varphi_{2}$ = 45° section of Euler space. The red arrows indicate intraband orientation evolution from the {111} matrix toward the <110>//ND fiber.

Before calculating ω, the boundaries of the <111>//ND matrix orientation region must be defined. The rolling texture of BCC metals is concentrated on the $\varphi_{2}$ = 45° section of Euler space. The Taylor factor distribution on this section is shown in Figure 2. Barnett [42] proposed that a Taylor factor ≥ 3.4 can serve as the criterion for a matrix to trigger shear bands. Figure 2 shows that orientations meeting this criterion lie mainly within 40° ≤ Φ ≤ 70°. Since the interval 60° ≤ $\varphi_{1}$ ≤ 90° already covers a complete <111>//ND unit ({111}<110>–{111}<112>), the region bounded by 40° ≤ Φ ≤ 70° and 60° ≤ $\varphi_{1}$ ≤ 90° (shaded rectangle in Figure 2) was taken as the representative matrix orientation region for analyzing ω. Orientations within this region are hereafter referred to as the {111} matrix.

During the intraband shear deformation, the dominant feature of the orientation evolution is a transition from the <111>//ND fiber towards the <110>//ND fiber, corresponding to a monotonic increase in Φ (arrows in Figure 2). The rate of change of Φ is therefore adopted here to represent the intraband lattice rotation rate ω. From Equation (12), and noting that $\Omega_{13}^{*}$ = $-\Omega_{31}^{*}$:

(14) $$\omega =\dot{\Phi }=c\left(\Omega_{13}^{*}sin\varphi_{1}-\Omega_{23}^{*}cos\varphi_{1}\right),$$

where c = 180/π is a constant, $\varphi_{1}$ is the instantaneous Euler angle component, and $\Omega_{23}^{*}$ and $\Omega_{13}^{*}$ are components of the lattice spin rate $\Omega^{*}$. A positive ω indicates rotation of the lattice towards the <110>//ND fiber, whereas a negative ω indicates rotation in the opposite sense.

Calculations over the above <111>//ND matrix orientation region show that only nine slip systems are active during the intraband shear deformation. These nine can be divided into two groups according to whether their contributions suppress or promote the increase in Φ, as listed in Table 2.

## 3. Retardation Plane and Intraband Lattice Rotation

### 3.1. Relationship Between the Shear Band Angle and the Position of the Retardation Plane

**Figure 3:**
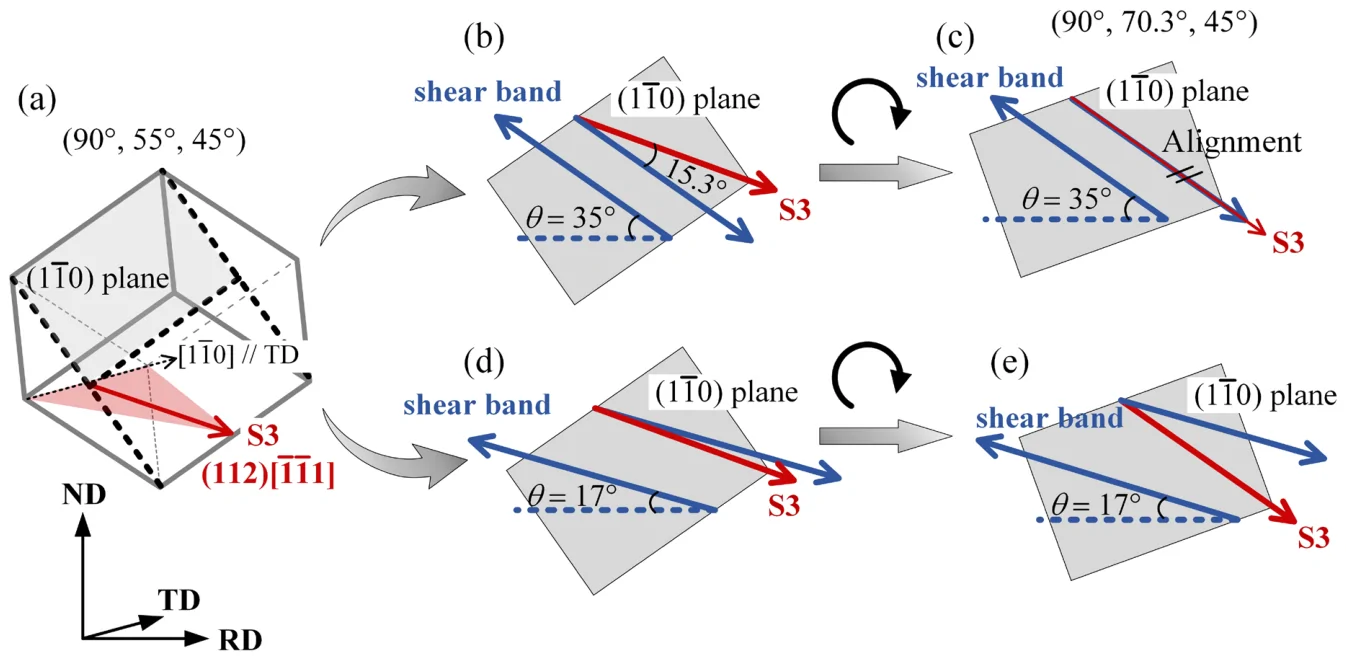
Geometric relationship between the trace of slip system S3 and the shear band for (90°, 55°, 45°). (**a**) Configuration of S3 in the lattice of (90°, 55°, 45°); (**b**,**c**) S3 relative to a θ = 35° shear band before and after a lattice rotation of 15.3°; (**d**,**e**) S3 relative to a θ = 17° shear band before and after lattice rotation.

Murakami et al. [43] reported that, for orientations lying on the $\left[1\bar{1}0\right]$//TD fiber within the {111} matrix region, the lattice rotation can be completely retarded by slip system S3 $\left(112\right)\left[\bar{1}\bar{1}1\right]$, giving rise to ω = 0. Figure 3 illustrates this mechanism using (90°, 55°, 45°), a representative $\left[1\bar{1}0\right]$//TD orientation within the {111} matrix (Figure 3a). In the lattice of this orientation, both the slip direction and the slip plane normal of S3 lie in the $\left(1\bar{1}0\right)$ plane, as does the shear direction. The three-dimensional relative rotation between S3 and the shear direction can therefore be represented by a two-dimensional rotation of the $\left(1\bar{1}0\right)$ plane about the TD axis. The intraband shear deformation rotates the lattice clockwise. For a θ = 35° shear band (Figure 3b), the band lies ahead of S3 along this rotation path. After a lattice rotation of 15.3° the intraband orientation reaches (90°, 70.3°, 45°) (Figure 3c), at which point the trace of S3 coincides exactly with the shear band. When the Schmid factor of S3 reaches its maximum value of 1.0, the plastic spin generated by S3 fully cancels the spin imposed by the shear band, thereby stopping lattice rotation.

When the reasoning of Murakami et al. [43] is extended to shear bands of lower angle, the retarding effect of S3 on the lattice rotation no longer always holds. Taking a θ = 17° shear band as an example, S3 already lies ahead of the band along the clockwise rotation path at the onset of shear deformation (Figure 3d). As the lattice rotates, S3 therefore moves progressively further away from the shear direction (Figure 3e), so that it never has the opportunity to coincide with the shear band.

On the basis of the above mechanism, the range of matrix orientations subject to retardation by S3 at different values of θ can be derived from the slip geometry. Orientations on the $\left[1\bar{1}0\right]$//TD fiber all take the Euler angle form (90°, Φ, 45°), so that their Miller indices $\left[G\right]$ can be written as

(15) $$\left[G\right]=\left[\begin{array}{ccc} -cos\Phi & 1 & sin\Phi \\ -cos\Phi & -1 & sin\Phi \\ \sqrt{2}sin\Phi & 0 & \sqrt{2}cos\Phi \end{array}\right].$$

Since S3 operates in the negative sense, its slip direction b in the global coordinate system is

(16) $$b={\left[G\right]}^{T}\cdot \left[\begin{array}{c} 1\\ 1\\ -1 \end{array}\right]=\left[\begin{array}{c} -2cos\Phi -\sqrt{2}sin\Phi \\ 0\\ 2sin\Phi -\sqrt{2}cos\Phi \end{array}\right],$$

whose magnitude is invariably $\sqrt{6}$. Normalizing b and noting that $2/\sqrt{6}$ = cos35.3° and $\sqrt{2}/\sqrt{6}$ = sin35.3° gives

(17) $$\frac{b}{\left|b\right|}=\left[\begin{array}{c} -cos\left(\Phi -35.3{}^{\circ}\right)\\ 0\\ sin\left(\Phi -35.3{}^{\circ}\right) \end{array}\right].$$

Equation (17) shows that the angle between the slip direction of S3 and −RD is always Φ − 35.3°. This angle is determined solely by the lattice angle between <111> and <110> within the $\left(1\bar{1}0\right)$ plane. During the intraband shear deformation, the trace of S3 therefore approaches the shear band at the same rate as the lattice rotates.

For a shear band of angle θ, the shear direction $b^{sb}$ in the global coordinate system is

(18) $$b^{sb}=\left[\begin{array}{c} -cos\theta \\ 0\\ sin\theta \end{array}\right].$$

Since b and $b^{sb}$ both lie in the ND–RD plane and make angles of Φ − 35.3° and θ with −RD, respectively, the condition for the trace of S3 to coincide with the shear band is

(19) Φ−35.3°=θ.

For a given θ, the value of Φ satisfying this condition defines the position of the retardation plane, denoted $\Phi_{r}$:

(20) $$\Phi_{r}=35.3{}^{\circ}+\theta .$$

Since Φ increases monotonically during the intraband shear deformation, every matrix orientation with Φ ≤ $\Phi_{r}$ must encounter retardation by S3 in the course of its evolution. For the {111} region considered here (40° ≤ Φ ≤ 70°), matrix orientations free from S3 retardation begin to appear once θ falls below 34.7°.

It should be noted that the present analysis explicitly considers only the 60° ≤ $\varphi_{1}$ ≤ 90° orientation unit on the $\varphi_{2}$ = 45° section. Owing to rolling symmetry, another set of equivalent {111} matrix variants exists within the 30° ≤ $\varphi_{1}$ ≤ 60° orientation unit. For these variants, the shear bands that form within the matrix should exhibit a geometry mirror-symmetric about ND relative to that shown in Figure 1. This correspondence between the matrix orientation and the shear band configuration is determined by the orientation-softening condition for shear band formation. Accordingly, for these symmetry-related variants, a {112}<111> slip system crystallographically equivalent to S3 is expected to play the same dominant retarding role. Therefore, S3 should be regarded as the representative dominant retarding slip system for the symmetry unit analyzed in the present study.

### 3.2. Dependence of the Intraband Lattice Rotation Rate on the Shear Band Angle

**Figure 4:**
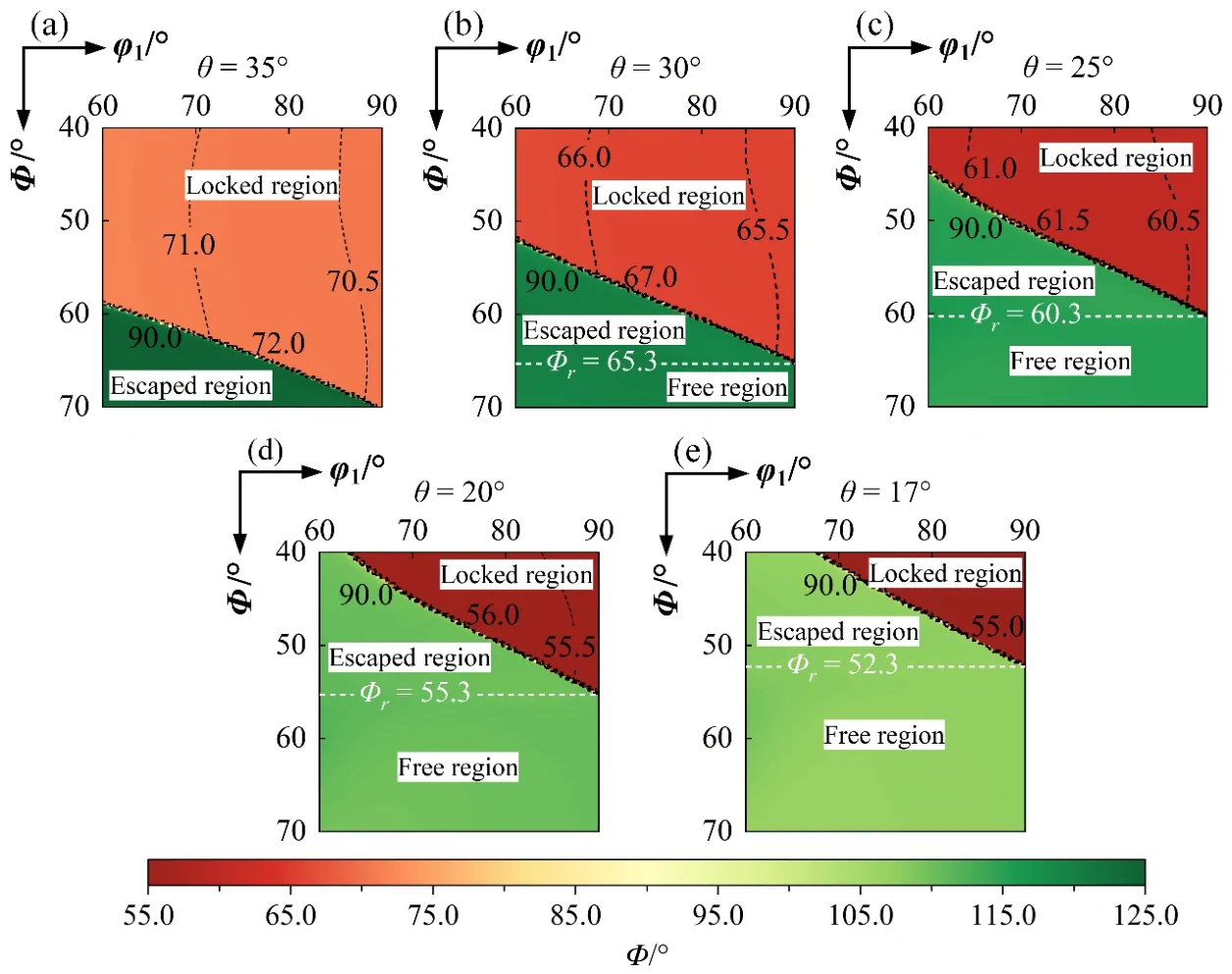
Intraband Φ values of the {111} matrix at a shear strain of 6.0, mapped onto the matrix orientation region for shear bands of different angles: (**a**) θ = 35°, (**b**) θ = 30°, (**c**) θ = 25°, (**d**) θ = 20° and (**e**) θ = 17°.

Previous studies [44] indicate that the maximum shear strain $\gamma^{sb}$ within shear bands in BCC iron-based alloys is about 6.0. Accordingly, whether the intraband orientation reaches the <110>//ND fiber (i.e., Φ = 90°) at this shear strain can be used as a measure of whether the retardation by S3 is overcome. The same finite-strain criterion was also adopted in our previous study to assess the intraband lattice rotation capacity [45]. To verify the slip geometry derivation in Section 3.1, the crystal plasticity model described in Section 2.2 was used. The intraband Φ values of the {111} matrix were calculated at a shear strain of 6.0 for five shear band angles: θ = 35°, 30°, 25°, 20° and 17°. These five angles were chosen because 35° and 17° are the frequently reported upper and lower values of θ, which together delimit the principal range of shear band angles in cold-rolled silicon steel. The intermediate angles of 30°, 25° and 20° were added to reveal the transition. Figure 4 presents the resulting Φ distributions mapped onto the orientation region of the {111} matrix. For every shear band angle, the intraband Φ distribution exhibits a steep gradient. The inclined Φ = 90° contour acts as a boundary that divides the {111} matrix region into two distinct parts. At a shear strain of 6.0, matrix orientations in the green region at the lower left have all crossed the <110>//ND fiber, whereas those in the red region at the upper right remain retarded within the {111} region. For matrix orientations on this boundary, the corresponding intraband orientations reach the <110>//ND fiber (Φ = 90°) at $\gamma^{sb}$ = 6.0. The theoretical retardation plane of the θ = 35° shear band ($\Phi_{r}$ = 70.3°) lies outside the {111} region. For the other four angles, the boundary intersects the $\left[1\bar{1}0\right]$//TD fiber at Φ = 65.3° (θ = 30°), 60.3° (θ = 25°), 55.3° (θ = 20°) and 52.3° (θ = 17°), in exact agreement with Equation (20). This confirms that the matrix orientations in the red region are indeed retarded by S3 during the intraband shear deformation.

The crystal plasticity results in Figure 4 nevertheless differ from the prediction of the slip geometry. The inclination of the boundary (the Φ = 90° contour) indicates that not all matrix orientations with Φ ≤ $\Phi_{r}$ are completely retarded by S3 within a finite shear strain. Accordingly, and as marked in Figure 4, the part of the interval Φ ≤ $\Phi_{r}$ that reaches the <110>//ND fiber within a finite shear strain is hereafter termed the escaped region, and the part that fails to reach it is termed the locked region. The interval Φ > $\Phi_{r}$, which is theoretically free from S3 retardation, is termed the free region.

The shear band angle has a pronounced influence on the intraband lattice rotation behavior of the {111} matrix. Comparison of the panels in Figure 4 shows that, as θ decreases, the proportion of the locked region within the {111} region decreases while that of the free region increases. This trend follows directly from Equation (20). More noteworthy is the influence of the shear band angle on the escaped region, which manifests itself in two respects. First, the proportion of the escaped region within the {111} region rises progressively as θ decreases: 20.3% (θ = 35°), 22.5% (θ = 30°), 24.5% (θ = 25°), 24.8% (θ = 20°) and 25.4% (θ = 17°). Second, the intraband lattice rotation rate ω of the escaped region increases as θ decreases.

**Figure 5:**
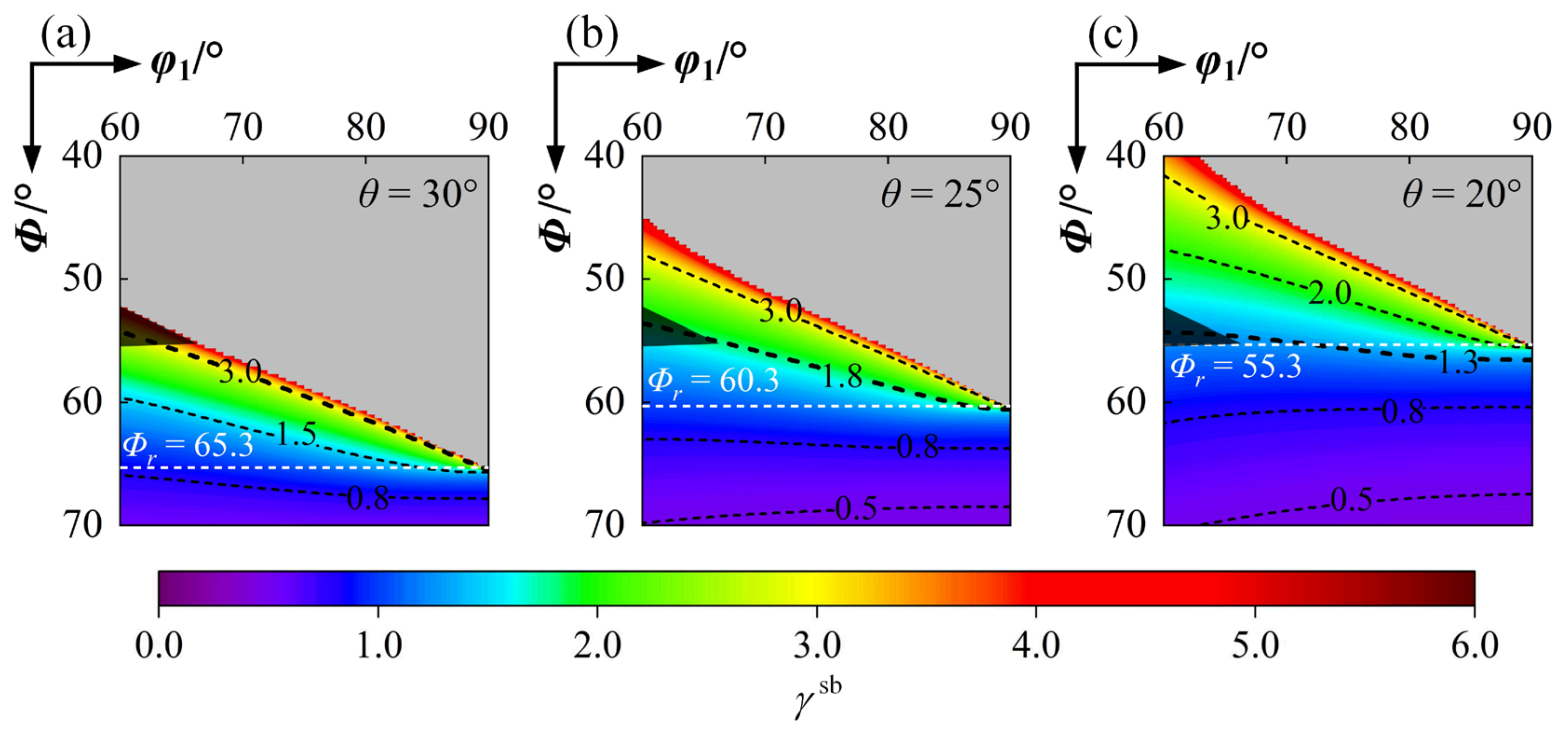
Shear strain $\gamma^{sb}$ required for the {111} matrix to reach the <110>//ND fiber during intraband shear deformation: (**a**) θ = 30°, (**b**) θ = 25° and (**c**) θ = 20°. The gray area indicates orientations that fail to reach the <110>//ND fiber within $\gamma^{sb}$ = 6.0.

Figure 5 shows the shear strain $\gamma^{sb}$ required for the {111} matrix region to reach the <110>//ND fiber during intraband shear deformation at θ = 30°, 25° and 20°. A lower $\gamma^{sb}$ required to reach the <110>//ND fiber indicates a higher overall intraband lattice rotation rate. The black shaded area denotes the escaped region common to all three shear band angles. Within this region, $\gamma^{sb}$ falls from about 3.0 at θ = 30° (Figure 5a) to about 1.8 at θ = 25° (Figure 5b), and further to about 1.3 at θ = 20° (Figure 5c). Since matrix orientations in the free region are not retarded by S3 during their intraband evolution, they require a lower shear strain than those in the escaped region to rotate to the <110>//ND fiber. Unlike the escaped region, however, the $\gamma^{sb}$ of the free region is little affected by θ. Why the escaped region arises, and why both its proportion and its intraband lattice rotation rate ω vary with θ, are the key questions addressed by the quasi-in situ verification and the discussion that follow.

## 4. Quasi-in-Situ Experimental Verification

### 4.1. Selection of the Target Grain

**Figure 6:**
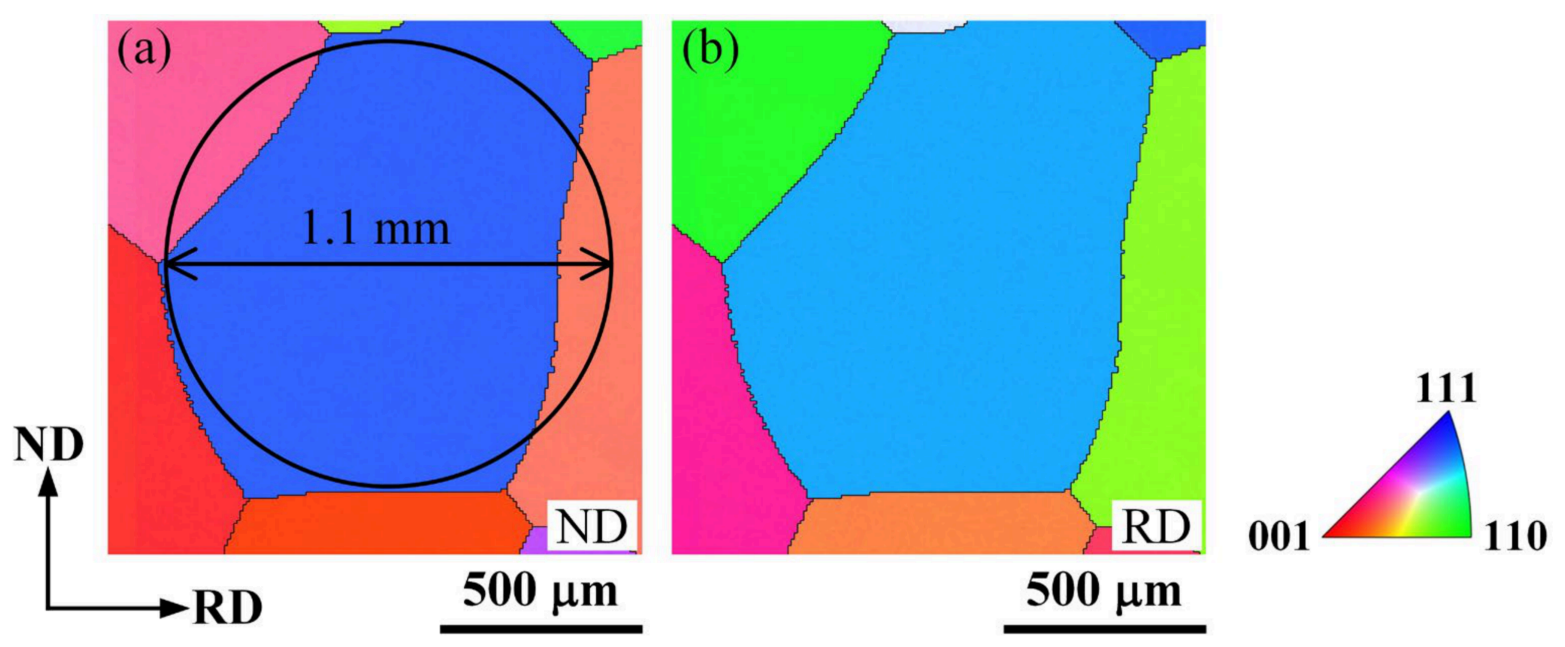
IPF maps of the initial target grain: (**a**) ND-IPF and (**b**) RD-IPF.

To ensure that the shear band angle θ was the sole variable, the quasi-in situ tracking was confined to a single grain, so that the matrix state remained identical for all shear bands examined. The target grain had an initial orientation of (85.9°, 44.2°, 34.1°), with a misorientation of about 15° from the ideal Copper orientation (90°, 35°, 45°). During the uniform deformation stage preceding shear band initiation, this orientation converges towards the {111} region from the low-Φ side, thereby providing a matrix that satisfies the condition for shear band formation. Figure 6 shows the inverse pole figure (IPF) maps of the initial grain. Its equivalent circle diameter reaches 1.1 mm, which is large enough to accommodate shear bands of several angles within one grain while retaining sufficient area under plane strain compression.

### 4.2. Matrix Orientation and Its Corresponding Region

**Figure 7:**
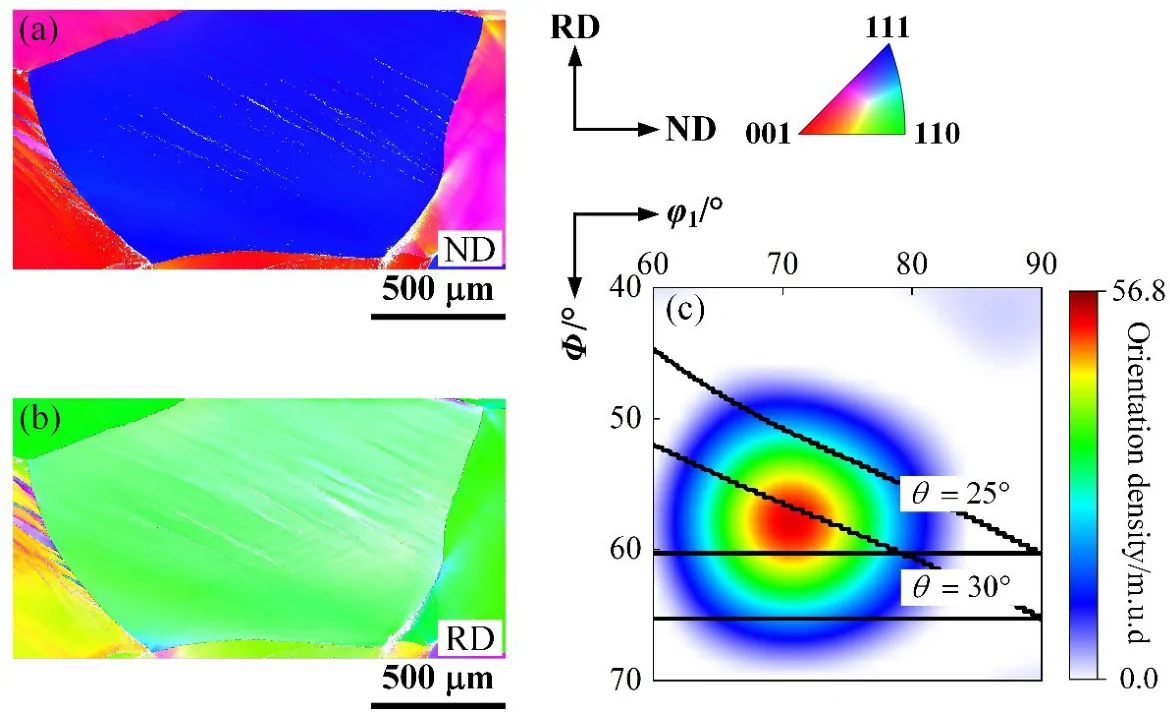
Morphology and orientation of the target grain at a cumulative true strain of 0.51: (**a**) ND-IPF map, (**b**) RD-IPF map, (**c**) ODF on the $\varphi_{2}$ = 47° section with the region boundaries for θ = 30° and 25° shear bands superimposed.

The initial grain was rolled in 20 passes to a cumulative true strain of 0.51 (40% reduction); the resulting matrix morphology and orientation are shown in Figure 7. The IPF color maps (Figure 7a,b) show that the matrix now lies close to {111}<110>, with its orientation maximum at (71.9°, 58.0°, 47.1°), confirming that the target grain had converged towards {111} during the early stage of rolling as expected. Since the orientation maximum lies on the $\varphi_{2}$ = 47° section, the orientation distribution function (ODF) of the target grain is presented on this section in Figure 7c. The boundaries separating the locked, escaped and free regions for θ = 30° and 25° shear bands, recalculated for this section, are superimposed on the ODF. The present matrix orientation falls in the escaped region for both angles, where ω is most sensitive to θ.

### 4.3. Intraband Orientation Distribution in Shear Bands of Different Angles

**Figure 8:**
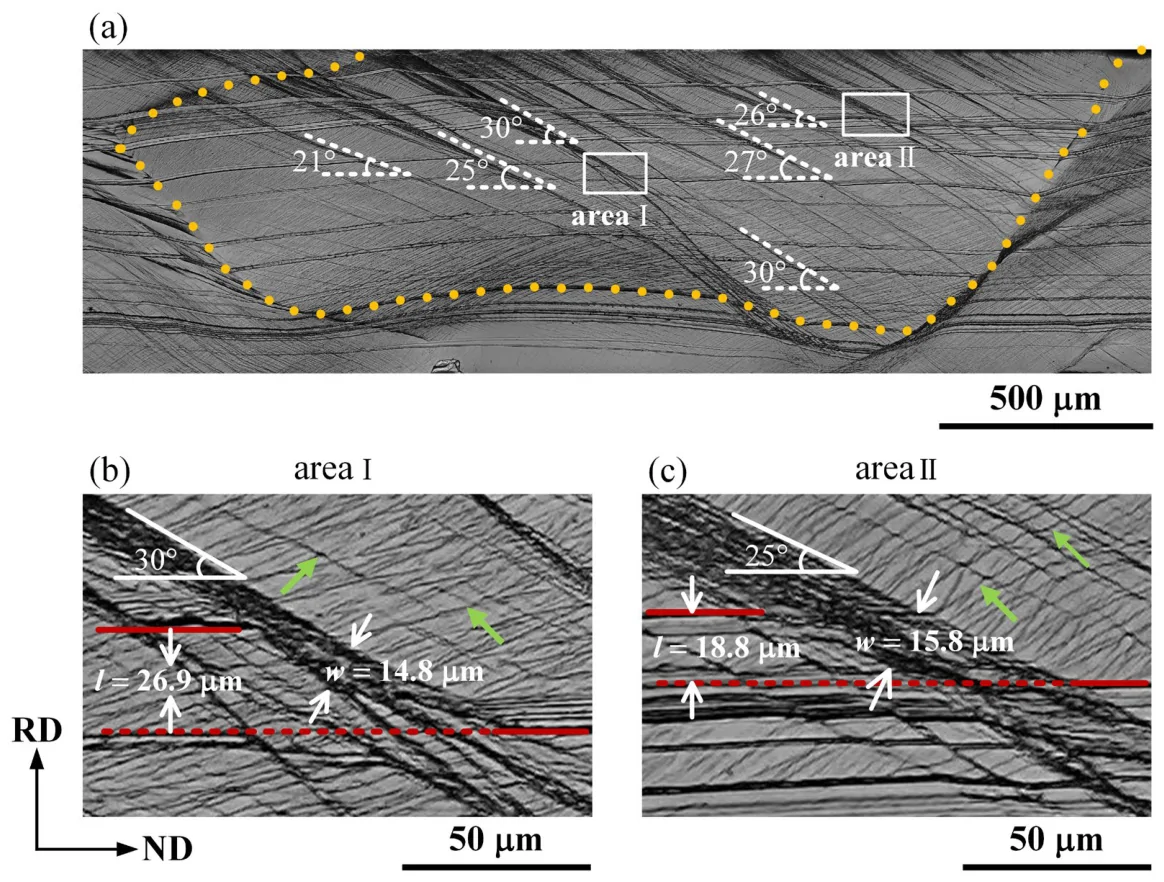
Metallographic morphology of the target grain at a cumulative true strain of 0.8: (**a**) overall morphology; (**b**) area I on the θ = 30° shear band; (**c**) area II on the θ = 25° shear band. The green arrows in (**b**,**c**) indicate representative slip traces in the matrix.

The target grain was rolled again in a single pass to a cumulative true strain of 0.8 (55% reduction) in order to promote the formation of numerous shear bands. The metallographic morphology of the target grain after this rolling is shown in Figure 8. The yellow dotted line in Figure 8a outlines the grain boundary of the target grain. Shear bands have formed extensively within the grain and traverse it completely, with angles mostly between 20° and 30°. Two shear bands with clearly measurable scratch displacements, θ = 30° and θ = 25°, were selected for closer examination. Figure 8b and Figure 8c show enlarged views of areas I (from the θ = 30° shear band) and II (from the θ = 25° shear band), as marked in Figure 8a. The solid and dashed red lines indicate the positions of the pre-introduced scratch on either side of the shear band; l is the component of the scratch displacement along ND, and w is the width of the shear band. The shear strain $\gamma^{sb}$ carried by the shear band is then calculated as

(21) $$\gamma^{sb}=\frac{l}{w\cdot sin\theta }.$$

Substituting the values measured in Figure 8b,c gives shear strains of 3.63 and 2.82 for the θ = 30° and θ = 25° shear bands, respectively. It should also be noted that fine lines inclined to RD, as indicated by the green arrows in Figure 8b,c, are slip traces generated by matrix deformation during rolling. These traces are not involved in the shear-strain measurement and are therefore not discussed further.

**Figure 9:**
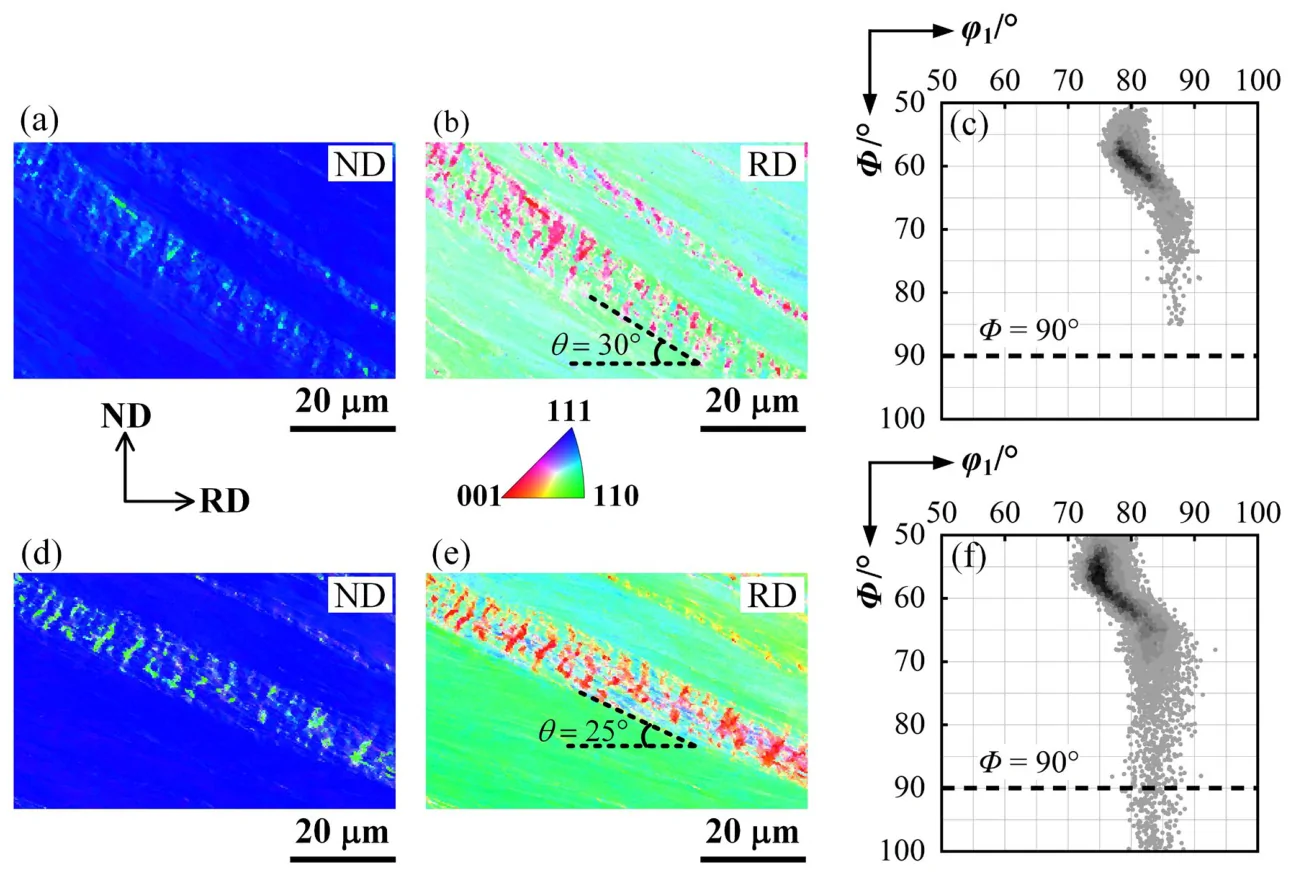
Intraband orientations of the θ = 30° (top row) and θ = 25° (bottom row) shear bands: (**a**,**d**) ND-IPF maps, (**b**,**e**) RD-IPF maps and (**c**,**f**) scatter plots of the intraband orientations on the $\varphi_{2}$ = 45° section. Darker scatter indicates higher point density.

The intraband orientations of these two shear bands were then measured to examine the influence of θ on the intraband lattice rotation. The results are shown in Figure 9. Comparison of the ND-IPF maps shows that the <110>//ND areas within the θ = 25° band (Figure 9d) are denser than those within the θ = 30° band (Figure 9a). Figure 9c,f present the orientations of all pixels within the two bands as scatter plots on the $\varphi_{2}$ = 45° section. In both bands, the orientations extend from Φ ≈ 58° towards the <110>//ND fiber. This starting point coincides with the Φ value of the matrix orientation maximum. The shape of the spread path is likewise identical, indicating that both bands originated from a matrix in the same state. The extent of the orientation evolution, however, differs markedly: the leading edge of the scatter within the θ = 30° band has not yet reached the <110>//ND fiber, whereas that within the θ = 25° band has already crossed it and extends to Φ = 100°. Notably, the θ = 30° band carries a considerably larger shear strain (3.63) than the θ = 25° band (2.82), yet achieves a smaller lattice rotation despite this larger strain. The θ = 25° band therefore possesses a higher intraband lattice rotation rate. This agrees with the calculated prediction that shear bands of lower angle are more favorable for the intraband orientation of the escaped region matrix to develop towards the <110>//ND fiber.

## 5. Discussion

### 5.1. Slip System Competition and the Origin of the Escaped Region

**Figure 10:**
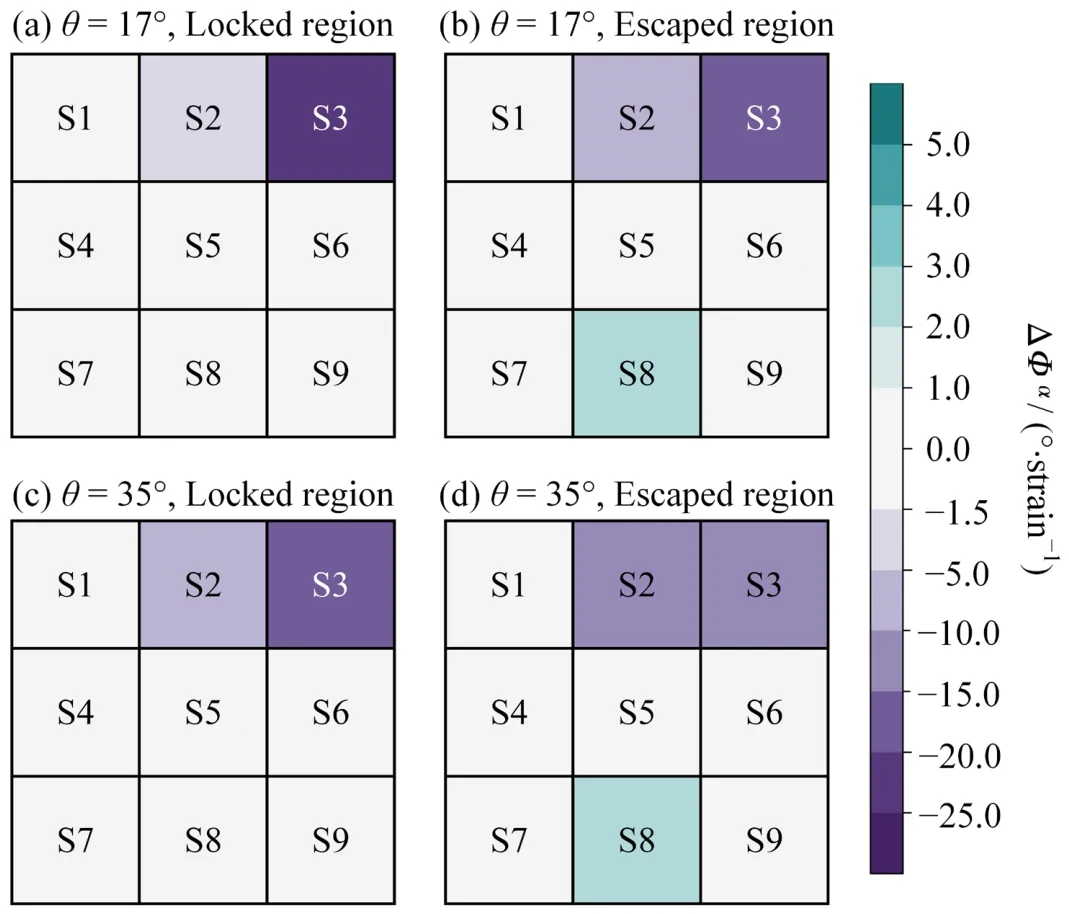
Contribution of slip system α to the change in Φ per unit intraband shear strain, $\Delta \Phi^{\alpha }$, as the intraband orientation crosses the retardation plane. The values are averaged over all orientations in the corresponding region: (**a**,**b**) θ = 17° and (**c**,**d**) θ = 35°.

As shown by the slip geometry analysis in Section 3.1, the key factor governing the lattice rotation rate is the retarding effect of slip system S3 $\left(112\right)\left[\bar{1}\bar{1}1\right]$, which reaches its maximum at the retardation plane. Analyzing the contribution of each slip system as the intraband orientation crosses the retardation plane is therefore essential to understanding the origin of the lattice rotation rate. Figure 10 shows the contribution of slip system α to the change in Φ per unit intraband shear strain, denoted as $\Delta \Phi^{\alpha }$, as the intraband orientation of the {111} matrix crosses the retardation plane. $\Delta \Phi^{\alpha }$ > 0 indicates that the plastic spin generated by slip system α contributes to an increase in Φ, thereby promoting lattice rotation toward the <110>//ND fiber. In contrast, $\Delta \Phi^{\alpha }$ < 0 indicates that the slip system opposes the increase in Φ, thereby retarding lattice rotation toward this fiber. Two angles are taken as examples: 17° and 35°, the lower and upper limits of the θ range considered here. The slip systems that significantly control the change in Φ during this crossing are the same for both angles. In the locked region, besides the primary slip system S3, the other retarding slip system S2 $\left(101\right)\left[\bar{1}\bar{1}1\right]$ also plays a key role in the change in Φ (Figure 10a,c). The escaped region differs distinctly: in addition to S3 and S2, the promoting slip system S8 $\left(1\bar{2}1\right)\left[111\right]$ exerts a comparably strong influence on Φ (Figure 10b,d). At the retardation plane, $\Phi_{r}=35.3{}^{\circ}+\theta$, so the angular relationship between the lattice and the shear band remains essentially unchanged as θ varies. Consequently, the same slip systems govern the change in Φ at the retardation plane for all shear-band angles considered. This simplifies the subsequent analysis: the influence of θ on ω can be treated as the outcome of the competition among the three slip systems S3, S2 and S8.

The slip geometry derivation in Section 3.1 and the results in Figure 10 together account for the origin of the escaped region as follows: When the {111} matrix orientation does not lie exactly on the $\left[1\bar{1}0\right]$//TD fiber, the slip plane trace of S3 cannot coincide fully with the shear band and thus loses its extreme orientation advantage. Part of the shear deformation originally carried by S3 is then taken up by other slip systems, mainly S2 and S8. Consequently, once S8 operates sufficiently, the plastic spin it generates counteracts the retarding effect of S3 and S2, allowing for the intraband orientation to overcome the retardation within a finite shear strain and reach the <110>//ND fiber.

### 5.2. Influence of the Shear Band Angle on the Slip System Competition

Two conditions must be met for ω to increase as the intraband orientation crosses the retardation plane. First, the activity of the primary slip system S3 must be low enough to release a sufficient share of the shear deformation to the secondary slip systems S2 and S8. Second, the activity of S8 relative to that of S2 must be high enough to minimize the cancellation of the imposed spin by the plastic spin. On this basis, the variation of ω in the escaped region matrix with the shear band angle θ can be analyzed further.

Assuming that the stress acting on the intraband orientation at the retardation plane is coaxial with the shear strain, its directional component ${\hat{\tau }}^{sb}$ follows from the shear-band geometry (Figure 1) as

(22) $${\hat{\tau }}^{sb}=\left[\begin{array}{ccc} sin2\theta & 0 & cos2\theta \\ 0 & 0 & 0\\ cos2\theta & 0 & -sin2\theta \end{array}\right].$$

An intraband orientation that crosses the retardation plane necessarily has Φ = $\Phi_{r}$ at that instant and can therefore be written as $\left(\varphi_{1},{\Phi_{r},\varphi }_{2}\right)$ with $\varphi_{1}$ and $\varphi_{2}$ retaining their instantaneous values. Substituting $\Phi_{r}=35.3{}^{\circ}+\theta$, the resolved shear stress of S3 along this stress direction, ${\hat{\tau }}^{\left(3\right)}$, becomes

(23) $$\begin{array}{c} {\hat{\tau }}^{\left(3\right)}=\frac{{\left(1+sin\varphi_{1}\right)}^{2}}{36}\left[6+2sin2\varphi_{2}+\frac{\left(sin\varphi_{2}+cos\varphi_{2}\right)}{\sqrt{2}}\right]\\ +cos\varphi_{1}\sin\left(\varphi_{2}-45{}^{\circ}\right)\cos\left(\theta +35.3{}^{\circ}\right). \end{array}$$

According to Equation (4), the operation of a slip system scales with the m-th power (m = 20) of its resolved shear stress. The orientation activity of S3 is therefore defined as

(24) $$A_{o}^{\left(3\right)}={\left[{\hat{\tau }}^{\left(3\right)}\right]}^{m}.$$

**Figure 11:**
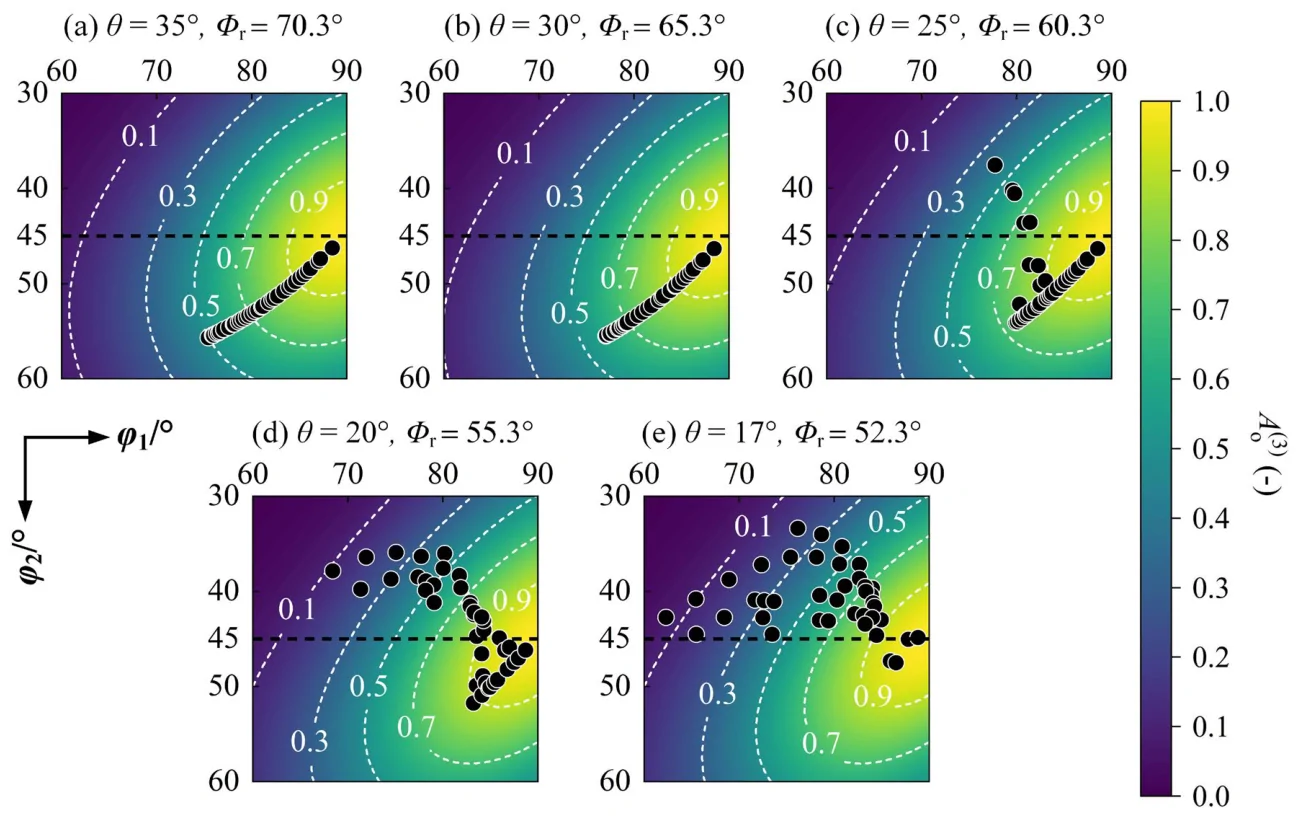
Distribution of $A_{o}^{\left(3\right)}$ on the retardation planes for shear bands of different angles: (**a**) θ = 35°, (**b**) θ = 30°, (**c**) θ = 25°, (**d**) θ = 20° and (**e**) θ = 17°. Black dots mark the positions at which the 40 selected matrix orientations cross the corresponding retardation plane.

Equation (23) shows that θ appears only in the second term of ${\hat{\tau }}^{\left(3\right)}$, which carries $cos\varphi_{1}sin\left(\varphi_{2}-45{}^{\circ}\right)$ as its factor. For orientations with $\varphi_{1}$ = 90° or $\varphi_{2}$ = 45° this factor vanishes and the influence of θ disappears entirely. Away from these positions it remains a second-order small quantity with respect to the deviation from the $\left[1\bar{1}0\right]$//TD fiber. The color maps in Figure 11 confirm this directly: the $A_{o}^{\left(3\right)}$ distributions on the retardation planes Φ = $\Phi_{r}$ for θ = 35°, 30°, 25°, 20° and 17° are almost identical, with no appreciable change in the position or shape of the contours. θ therefore has only a marginal effect on the activity of S3 at the retardation plane.

In addition, 40 representative matrix orientations were selected to show the orientation distribution trend without obscuring the underlying color maps and contour lines. For each of the five shear band angles, the position at which these orientations cross the corresponding retardation plane was determined. The crossing positions are superimposed on the $A_{o}^{\left(3\right)}$ distributions in Figure 11. These orientations were taken from the escaped region of the θ = 17° shear band. Orientations in this region have relatively low Φ values and therefore encounter the retardation plane at all five angles, so that the comparison can be made on the same set of orientations. When crossing the retardation planes of the θ = 35° and θ = 30° bands (Figure 11a,b), the crossing positions are concentrated within 75° ≤ $\varphi_{1}$ ≤ 90° and $\varphi_{2}$ ≥ 45°, where $A_{o}^{\left(3\right)}$ is only slightly reduced. At θ = 25° the crossing positions begin to spread towards lower $\varphi_{1}$ and $\varphi_{2}$ (Figure 11c). This trend becomes more pronounced at θ = 20° and 17° (Figure 11d,e). These changes arise from the relative position of the orientation evolution path and the retardation plane. During shear deformation the intraband orientation of the {111} matrix evolves towards higher $\varphi_{1}$ and $\varphi_{2}$. $\Phi_{r}$ decreases with θ, so that the matrix orientation encounters the retardation plane earlier. Less shear strain has accumulated within the band at that point, so the intraband orientation retains lower $\varphi_{1}$ and $\varphi_{2}$ values at the crossing and consequently a lower $A_{o}^{\left(3\right)}$. For the matrix orientation of the experimental target grain (71.9°, 58.0°, 47.1°), $A_{o}^{\left(3\right)}$ is 0.86 at the θ = 30° retardation plane ($\Phi_{r}$ = 65.3°) but only 0.46 at the θ = 25° plane ($\Phi_{r}$ = 60.3°).

Following the same procedure as in Equations (23) and (24), the resolved shear stress ${\hat{\tau }}^{\left(2\right)}$ and orientation activity $A_{o}^{\left(2\right)}$ of slip system S2 at the retardation plane are obtained as

(25) $${\hat{\tau }}^{\left(2\right)}=\frac{\sqrt{3}{\left(1+sin\varphi_{1}\right)}^{2}}{72}\left[6+2sin2\varphi_{2}-2cos2\varphi_{2}+\sqrt{2}cos\varphi_{2}\right]+\frac{1}{2}cos\varphi_{1}sin\theta$$

(26) $$A_{o}^{\left(2\right)}={\left[{\hat{\tau }}^{\left(2\right)}\right]}^{m}$$

Those of slip system S8 are obtained in the same way:

(27) $$\begin{array}{ll} {\hat{\tau }}^{\left(8\right)}= & \frac{{\left(1+sin\varphi_{1}\right)}^{2}}{72}\left[6+2sin2\varphi_{2}+6cos2\varphi_{2}+\sqrt{2}\left(cos\varphi_{2}-2sin\varphi_{2}\right)\right]\\ & +\frac{1}{6}cos\varphi_{1}\left(2\sqrt{6}cos\theta -\sqrt{3}sin\theta \right), \end{array}$$

(28) $$A_{o}^{\left(8\right)}={\left[{\hat{\tau }}^{\left(8\right)}\right]}^{m}.$$

**Figure 12:**
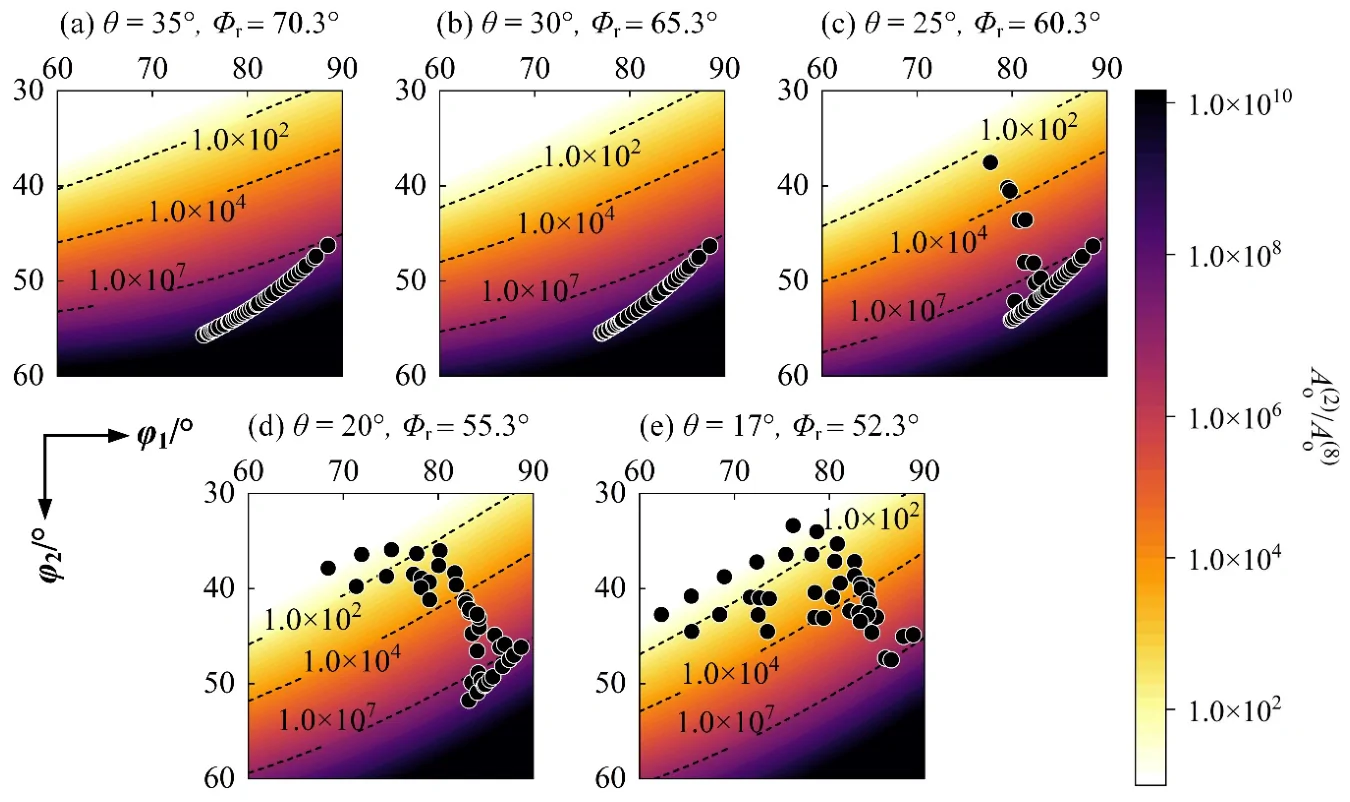
Distribution of $A_{o}^{\left(2\right)}$/$A_{o}^{\left(8\right)}$ on the retardation planes for shear bands of different angles: (**a**) θ = 35°, (**b**) θ = 30°, (**c**) θ = 25°, (**d**) θ = 20° and (**e**) θ = 17°. Black dots mark the positions at which the 40 selected matrix orientations cross the corresponding retardation plane.

Unlike ${\hat{\tau }}^{\left(3\right)}$, the θ-dependent terms in ${\hat{\tau }}^{\left(2\right)}$ and ${\hat{\tau }}^{\left(8\right)}$ both carry $cos\varphi_{1}$ as their factor, which is a first-order small quantity in (90° − $\varphi_{1}$). The influence of θ on $A_{o}^{\left(2\right)}$ and $A_{o}^{\left(8\right)}$ therefore cannot be neglected. Equations (25) and (27) show that, within 17° ≤ θ ≤ 35°, a decrease in θ reduces the θ-dependent term of ${\hat{\tau }}^{\left(2\right)}$ while increasing that of ${\hat{\tau }}^{\left(8\right)}$. The activity of S8 relative to S2 thus rises as θ decreases. Figure 12 presents the distribution of $A_{o}^{\left(2\right)}$/$A_{o}^{\left(8\right)}$ on the retardation planes for the five shear band angles. In agreement with the analytical result, the low-value region of $A_{o}^{\left(2\right)}$/$A_{o}^{\left(8\right)}$ (at low $\varphi_{1}$ and low $\varphi_{2}$) expands progressively as θ decreases. At the same time, the crossing positions of the {111} matrix orientations move towards this low-value region as θ decreases. For the matrix of the experimental target grain, $A_{o}^{\left(2\right)}$/$A_{o}^{\left(8\right)}$ falls from 4.4 × 10^6^ at θ = 30° to 4.6 × 10^3^ at θ = 25°.

In summary, a decrease in the shear-band angle raises the intraband lattice rotation rate for two reasons. First, a decrease in θ directly alters the distribution of slip system orientation activity on the retardation plane. Within 17° ≤ θ ≤ 35°, the orientation activity of the primary slip system S3 remains essentially unaffected by θ. However, the orientation activity of the rotation-promoting S8 relative to that of the rotation-retarding S2 increases as θ decreases. Second, a decrease in θ alters the distribution of the {111} matrix orientations at the crossing of the retardation plane. $\Phi_{r}$ decreases with θ, so that the intraband orientation encounters the retardation plane at a lower shear strain and its crossing positions converge towards lower $\varphi_{1}$ and $\varphi_{2}$. Crucially, this is the direction in which both $A_{o}^{\left(3\right)}$ and $A_{o}^{\left(2\right)}$/$A_{o}^{\left(8\right)}$ decrease: the former reduces the shear strain carried by S3 and leaves more room for the secondary slip systems to operate, while the latter further widens the advantage of S8 over S2.

### 5.3. Magnetic Implications and Applicability of the Present Analysis

The present study reveals the crystallographic mechanism by which the shear band angle θ affects the intraband lattice rotation rate ω. For grain-oriented silicon steel, a higher ω promotes the rotation of intraband orientations from the {111} matrix toward the <110>//ND fiber, thereby favoring a higher density of Goss areas within the shear bands. Although intraband Goss areas serve as key Goss seeds for secondary recrystallization, a higher intraband Goss density does not necessarily lead to better final magnetic properties. An insufficient density of intraband Goss areas may provide too few favorable nuclei for secondary recrystallization, whereas an excessive density may induce orientation pinning, thereby deteriorating the final magnetic properties. The present discussion is confined to the cold-rolling deformation stage, and what has been established is the relationship between θ and ω. The quantitative relationship between the intraband Goss density and the final magnetic properties remains to be verified by annealing experiments and magnetic measurements.

The analytical framework established in the present study is not limited to Fe–3%Si alloys, and its applicability mainly depends on the dominant plastic deformation mechanism of the material. The general slip geometry and crystal plasticity approach adopted here can be extended to BCC metals and high stacking fault energy FCC metals dominated by dislocation slip. Such extensions require adapting the active slip systems and material parameters. Meanwhile, composition-induced changes in slip system strength and hardening behavior may alter the quantitative response of intraband lattice rotation. For FCC metals with low stacking fault energy, such as Cu–Al alloys [46] and Cu–Zn alloys [47], deformation twinning is also an important plastic deformation mechanism. Paul et al. [48,49] reported that, within shear bands in pure Ag, a typical FCC metal with low stacking fault energy, certain twinning systems can exert a pronounced retarding effect on lattice rotation. Therefore, for such metals, the present analysis based on dislocation slip needs to be extended to incorporate twinning mechanisms.

## 6. Conclusions

This work combines slip geometry analysis, crystal plasticity calculations and quasi-in situ characterization to examine how the shear band angle θ governs the intraband lattice rotation rate of the {111} matrix (60° ≤ $\varphi_{1}$ ≤ 90°, 40° ≤ Φ ≤ 70°, $\varphi_{2}$ = 45°) and to clarify the underlying slip mechanism. The main conclusions are as follows:

(1) A quantitative relationship between the position of the retardation plane and the shear band angle was established. On the $\left[1\bar{1}0\right]$//TD fiber, the angle between the slip direction of the primary retarding slip system S3 $\left(112\right)\left[\bar{1}\bar{1}1\right]$ and −RD is invariably Φ − 35.3°, where 35.3° is the angle between <111> and <110> within the $\left(1\bar{1}0\right)$ plane. The position of the retardation plane, $\Phi_{r}$, is accordingly defined as the Φ value at which the trace of S3 coincides with the shear band: $\Phi_{r}$ = 35.3° + θ. Since Φ increases monotonically during the intraband shear deformation, every {111} matrix orientation with Φ ≤ $\Phi_{r}$ must encounter retardation by S3 in the course of its evolution. Once θ falls below 34.7°, matrix orientations free from S3 retardation begin to appear within the {111} region.

(2) The {111} matrix was divided into three regions according to whether the intraband orientation reaches the <110>//ND fiber within a shear strain of 6.0. The crystal plasticity calculations show that some orientations with Φ ≤ $\Phi_{r}$ are not completely retarded by S3. On this basis, the part of the interval Φ ≤ $\Phi_{r}$ that reaches the <110>//ND fiber is termed the escaped region and the part that fails to reach it the locked region. The interval Φ > $\Phi_{r}$, theoretically free from S3 retardation, is termed the free region. As θ decreases from 35° to 17°, the locked region contracts, the free region expands, and the proportion of the escaped region rises from 20.3% to 25.4%. The intraband lattice rotation rate ω of the free region is little affected by θ.

(3) The ω of the escaped region matrix rises as θ decreases, a trend confirmed by the quasi-in situ characterization. Within a single grain, the θ = 30° and 25° shear bands carried shear strains of 3.63 and 2.82, respectively. Although the θ = 30° band carried the larger strain, the leading edge of the scatter in the former had not yet reached the <110>//ND fiber, whereas that in the latter had already crossed it. The calculations attribute this influence of θ on ω to two effects. First, a decrease in θ lowers the activity of the retarding secondary slip system S2 $\left(101\right)\left[\bar{1}\bar{1}1\right]$ and raises that of the promoting slip system S8 $\left(1\bar{2}1\right)\left[111\right]$ on the retardation plane, while the activity of the primary slip system S3 remains essentially unaffected. Second, a decrease in θ reduces $\Phi_{r}$, so that the intraband orientation encounters the retardation plane at a lower shear strain. The crossing position then converges towards the direction in which S3 and S2 are less active and S8 more active.

## Figures and Tables

**Table 1:** Parameters used in the crystal plasticity calculations.

Parameter	Symbol	Value	Unit	Reference
Elastic stiffness constants	$C_{11},\;C_{12},\;C_{44}$	222, 135, 120	GPa	[37]
Initial slip strength	$g_{0}$	161	MPa	[38,39]
Saturation slip strength	$\tau_{s}$	1137	MPa	
Initial hardening modulus	$h_{0}$	60	MPa	[40]
Self- and latent-hardening interaction coefficients	$q,q_{1}$	1.0, 1.4	-	[38,41]
Rate sensitivity exponent	*m*	20	-	[38,40]
Reference slip rate	${\dot{\gamma }}^{0}$	0.001	$s^{-1}$	

**Table 2:** Active slip systems of the {111} matrix during intraband shear deformation and their classification.

Retarding Lattice Rotation		Promoting Lattice Rotation	
Slip System	Crystallographic Definition	Slip System	Crystallographic Definition
S1	$\left(011\right)\left[\bar{1}\bar{1}1\right]$	S5	$\left(101\right)\left[1\bar{1}\bar{1}\right]$
S2	$\left(101\right)\left[\bar{1}\bar{1}1\right]$	S6	$\left(110\right)\left[1\bar{1}\bar{1}\right]$
S3	$\left(112\right)\left[\bar{1}\bar{1}1\right]$	S7	$\left(0\bar{1}1\right)\left[111\right]$
S4	$\left(2\bar{1}1\right)\left[\bar{1}\bar{1}1\right]$	S8	$\left(1\bar{2}1\right)[111]$
		S9	$\left(211\right)\left[1\bar{1}\bar{1}\right]$

## Data Availability

The original contributions presented in this study are included in the article/Supplementary Materials. Further inquiries can be directed to the corresponding author.

## Supplementary Materials

The following supporting information can be downloaded at: https://www.mdpi.com/article/10.3390/ma19214465/s1, The detailed derivation of Equation (1) is provided in the accompanying supplementary file.
