# Supplementary material for: Microstructural Origin of Magnetic Properties in Grain-Oriented Silicon Steel: Shear Band Angle Control of Intraband Lattice Rotation
Source: Materials (Basel). 2026 Oct 23;19(21):4465. doi: 10.3390/ma19214465 (PMC13763795; doi:10.3390/ma19214465)
Supplement: Supplementary file 1 [file materials-19-04465-s001.zip › materials-4521693-supplementary.pdf]

Supplementary Materials

Microstructural Origin of Magnetic Properties in Grain-Oriented Silicon Steel: Shear-Band Angle Control of Intraband Lattice Rotation

Sifei Cheng, Yuhui Sha, Fang Zhang, Gengsheng Cao, Zhangchen Xue and Liang Zuo

---

Kinematic Basis of Equation (1)

In finite-deformation crystal plasticity, for a general externally imposed deformation, the total deformation gradient $\mathbf{F}^{\mathrm{ext}}$ can be multiplicatively decomposed into the lattice and plastic parts:

$$\mathbf{F}^{\mathrm{ext}} = \mathbf{F}^{*}\mathbf{F}^{\mathrm{p}}. \tag{S1}$$

Here, $\mathbf{F}^{*}$ represents the lattice deformation and rotation, while $\mathbf{F}^{\mathrm{p}}$ represents the plastic deformation produced by crystallographic slip. The corresponding total velocity gradient is

$$\mathbf{L}^{\mathrm{ext}} = \dot{\mathbf{F}}^{\mathrm{ext}}(\mathbf{F}^{\mathrm{ext}})^{-1}. \tag{S2}$$

Differentiating the multiplicative decomposition gives

$$\mathbf{L}^{\mathrm{ext}} = \dot{\mathbf{F}}^{*}(\mathbf{F}^{*})^{-1} + \mathbf{F}^{*}\dot{\mathbf{F}}^{\mathrm{p}}(\mathbf{F}^{\mathrm{p}})^{-1}(\mathbf{F}^{*})^{-1}. \tag{S3}$$

Thus, the total velocity gradient $\mathbf{L}^{\mathrm{ext}}$ associated with the imposed deformation is accommodated jointly by the lattice and plastic contributions. In the current (global) coordinate system used in the present calculation, these two contributions are denoted as

$$\mathbf{L}^{*} = \dot{\mathbf{F}}^{*}(\mathbf{F}^{*})^{-1}, \tag{S4}$$

and

$$\mathbf{L}^{\mathrm{p}} = \mathbf{F}^{*}\dot{\mathbf{F}}^{\mathrm{p}}(\mathbf{F}^{\mathrm{p}})^{-1}(\mathbf{F}^{*})^{-1}, \tag{S5}$$

respectively. Therefore,

$$\mathbf{L}^{\mathrm{ext}} = \mathbf{L}^{*} + \mathbf{L}^{\mathrm{p}}. \tag{S6}$$

Each velocity gradient can be uniquely decomposed into its symmetric and antisymmetric parts:

$$\mathbf{L} = \mathbf{D} + \mathbf{\Omega}. \tag{S7}$$

Combining Equations (S6) and (S7) gives

$$\mathbf{D}^{\mathrm{ext}} + \mathbf{\Omega}^{\mathrm{ext}} = \mathbf{D}^{\mathrm{p}} + \mathbf{D}^{*} + \mathbf{\Omega}^{\mathrm{p}} + \mathbf{\Omega}^{*}. \tag{S8}$$

Separately equating the symmetric and antisymmetric parts, we obtain

$$\mathbf{D}^{\mathrm{ext}} = \mathbf{D}^{\mathrm{p}} + \mathbf{D}^{*}, \qquad \mathbf{\Omega}^{\mathrm{ext}} = \mathbf{\Omega}^{\mathrm{p}} + \mathbf{\Omega}^{*}. \tag{S9}$$

In the present study, the externally imposed deformation is the simple-shear deformation within a shear band. We therefore replace the superscript "ext" with "sb", giving

$$\mathbf{D}^{\mathrm{sb}} = \mathbf{D}^{\mathrm{p}} + \mathbf{D}^{*}, \qquad \mathbf{\Omega}^{\mathrm{sb}} = \mathbf{\Omega}^{\mathrm{p}} + \mathbf{\Omega}^{*}. \tag{S10}$$

Equation (S10) is identical to Equation (1) in the main manuscript. The superscript "sb" denotes the simple-shear deformation imposed within a shear band.
